# *CTNNB1* mutations, *TERT* polymorphism and CD8+ cell densities in resected hepatocellular carcinoma are associated with longer time to recurrence

**DOI:** 10.1186/s12885-022-09989-0

**Published:** 2022-08-13

**Authors:** Filip Ambrozkiewicz, Andriy Trailin, Lenka Červenková, Radka Vaclavikova, Vojtech Hanicinec, Mohammad Al Obeed Allah, Richard Palek, Vladislav Třeška, Ondrej Daum, Zbyněk Tonar, Václav Liška, Kari Hemminki

**Affiliations:** 1 Laboratory of Translational Cancer Genomics, Biomedical Center,Faculty of Medicine in Pilsen, Charles University, Alej Svobody 1665/76, 323 00 Pilsen, Czech Republic; 2 Laboratory of Cancer Treatment and Tissue Regeneration, Biomedical Center, Faculty of Medicine in Pilsen, Charles University, Prague, Czech Republic; 3 Department of Pathology, Third Faculty of Medicine, Charles University, Ruská 87, 100 00, Prague, 10 Czech Republic; 4 Laboratory of Pharmacogenomics, Biomedical Center, Faculty of Medicine in Pilsen, Charles University, Pilsen, Czech Republic; 5 Toxicogenomics Unit, National Institute of Public Health in Prague, Prague, Czech Republic; 6 Department of Surgery, Faculty of Medicine in Pilsen, Charles University, Alej 16 Svobody 80, 323 00 Pilsen, Czech Republic; 7 Sikl’s Institute of Pathology, Faculty of Medicine and Teaching Hospital in Plzen, Charles University, Plzen, Czech Republic; 8 Bioptická laboratoř s.r.o., Mikulášské nám, 4, 326 00 Pilsen, Czech Republic; 9 Department of Histology and Embryology, Faculty of Medicine in Pilsen, Charles University, Karlovarska 48, 301 66 Pilsen, Czech Republic; 10 Laboratory of Quantitative Histology, Biomedical Center, Faculty of Medicine in Pilsen, Charles University, Alej Svobody 1665/76, 323 00 Pilsen, Czech Republic; 11 Department of Cancer Epidemiology, German Cancer Research Center, Im Neuenheimer Feld 280, 69120 Heidelberg, Germany

**Keywords:** β-Catenin, TERT promoter, CD8+ cells, rs2853669, Hepatocellular carcinoma

## Abstract

**Background:**

Hepatocellular carcinoma (HCC) is a fatal disease characterized by early genetic alterations in telomerase reverse transcriptase promoter (TERTp) and β-catenin (CTNNB1) genes and immune cell activation in the tumor microenvironment. As a novel approach, we wanted to assess patient survival influenced by combined presence of mutations and densities of CD8+ cytotoxic T cells.

**Methods:**

Tissue samples were obtained from 67 HCC patients who had undergone resection. We analysed CD8+ T cells density, TERTp mutations, rs2853669 polymorphism, and CTNNB1 mutations. These variables were evaluated for time to recurrence (TTR) and disease free survival (DFS).

**Results:**

TERTp mutations were found in 75.8% and CTNNB1 mutations in 35.6% of the patients. TERTp mutations were not associated with survival but polymorphism rs2853669 in TERTp was associated with improved TTR and DFS. CTNNB1 mutations were associated with improving TTR. High density of CD8+ T-lymphocytes in tumor center and invasive margin correlated with longer TTR and DFS. Combined genetic and immune factors further improved survival showing higher predictive values. E.g., combining CTNNB1 mutations and high density of CD8+ T-lymphocytes in tumor center yielded HRs of 0.12 (0.03–0.52), *p* = 0.005 for TTR and 0.25 (0.09–0.74), *p* = 0.01 for DFS.

**Conclusion:**

The results outline a novel integrative approach for prognostication through combining independent predictive factors from genetic and immune cell profiles. However, larger studies are needed to explore multiple cell types in the tumor microenvironment.

**Supplementary Information:**

The online version contains supplementary material available at 10.1186/s12885-022-09989-0.

## Background

Primary livers cancer are the sixth most frequent diagnosed tumors globally and the third cause of cancer related death worldwide. Hepatocellular carcinoma (HCC) encompasses up to 85% of all primary liver cancers [1]. It develops as a result of a chronic liver disease which stepwise progresses from chronic inflammation to cirrhosis, fibrosis and HCC [2]. Malignant transformation of hepatocytes is a complex molecular pro-cess comprising numerous genetic and epigenetic alterations. These changes increase genetic diversity and accelerate evolution of tumor cells. However, increasing diversity stimulates immune system to action against cells presenting neoepitopes [3]. CD8+ cytotoxic T cells play a pivotal role in antitumor immune response. They are also one of many tumor-infiltrating lymphocytes (TIL), which are vital in predicting clinical outcome (overall survival (OS) and disease free-survival (DFS)) in primary and metastatic liver cancer. Evidence shows that high densities of CD8+ cells in HCC are associated with better OS and DFS [4]. These at-tributes point to the role of intratumor CD8+ cells as a potential prognostic biomarker [4, 5].

In cancer cells, reactivation of telomerase prevents telomere shortening and may lead to unlimited cellular proliferation, enabling subsequent transformation. Almost 90% of human cancers show reactivation of telomerase [6]. Telomerase reverse transcriptase (TERT) is the catalytic subunit of telomerase, which additionally contains the telomerase RNA component (TERC) [7]. In HCC, TERT promoter (TERTp) mutations are the most frequent and earliest somatic alterations with a prevalence of about 60% [8, 9]. Until to-date, two recurrent somatic mutations in TERTp (− 124 and − 146 upstream of the ATG start site) have been associated with many types of cancer, including HCC. They create de novo binding sites for the ETS family of transcription factors and increase TERT expression [10]. TERT single nucleotide polymorphism (SNP) rs2853669 disrupts an existing ETS2 binding site, which results in decreased telomerase activity [11]. It was shown that SNP rs2853669 correlates with patient survival with variable effects in various cancers [12–14]. On the other hand, the same SNP was associated with poor prognosis in HCC [15]. Hot-spot mutations present in exon 3 of the β-catenin (CTNNB1) gene are critical for hepatocarcinogenesis and are among the most frequent alterations (20–40% of cases) [16]. They predominantly occur at the phosphorylation sites, induce accumulation of nuclear β-catenin and lead to activation of the Wnt signaling pathway [17]. Moreover, mutations in exon 3 of CTNNB1tend to be associated with favourable prognosis in HCC [18, 19].

Research in the past decade has improved our understanding of hepatocarcinogenesis highlighting the multitude of genetic changes in the tumor but also the repertoire of interacting cells in the tumor microenvironments [20–22]. The aim of present study is to examine the interplay of most frequent driver mutations and one well-known immune marker. As a novel hypothesis, we posit that these two environments may act independently in influencing survival but when combined they may act additively supporting each other’s action. We chose to examine the interplay between genetic factors (TERTp and CTNNB1) in the tumor and immune factors (cytotoxic CD8+ cells) in the tumor microenvironment and their prognostic value in terms of time to recurrence (TTR), DFS and OS among resected HCC patients. Although the present patient series of 67 patients is relatively small it was homogenous as to the treatment by resection in a single center and precise follow-up thus providing a valuable addition to HCC patients.

## Methods

### Patient selection and characteristic

The study cohort consisted of 67 HCC patients (16 women and 51 men) of median age 69 years (range 24–86) who underwent primary tumor resection in Pilsen University hospital between 1997 and 2019. Patients were not subjected to neo-adjuvant therapy before operation, nor had distant metastasis. Clinical characteristics of the enrolled patients are presented in Table 1. The study was conducted in accordance with the Declaration of Helsinki and was approved by the Ethical Board of the Faculty of Medicine and University hospital in Pilsen (118/2021, March 11 2021).

**Table 1:** Clinical characteristics of recruited patients

Variable	n (%)
Gender (*n* = 67)	
Male	51 (76.1%)
Female	16 (23.9%)
Disease background(*n* = 67)	
Cirrhosis	14 (20.9%)
Hepatitis C	3 (4.5%)
Alcoholic steatohepatitis	7 (10.4%)
Cryptogenic chronic hepatitis	7 (10.4%)
Mixed etiology of hepatitis	9 (13.4%)
NAFLD/NASH	2 (3.0%)/15 (22.4%)
Unknown	10 (14.9%)
Tumor Size (*n* = 67)	
< 5 cm	27 (40.3%)
> 5 cm	34 (50.7%)
Unknown	4 (6.0%)
Tumor Number (*n* = 67)	
Solitary	56 (83.6%)
Multiple	8 (11.9%)
Unknown	3 (4.5%)
Tumor Stage (*n* = 67)	
I	45 (67.2%)
II	14 (20.9%)
III	5 (7.5%)
IV	3 (4.5%)
Event (*n* = 67)	
Recurrence	29 (43.3%)
Death	38 (56.7%)

### DNA extraction, sanger sequencing

DNA was extracted from FFPE (formaldehyde fixed paraffin embedded) tumor tissue with RecoverAll™ Total Nucleic Acid Isolation Kit for FFPE according to the manufacturer’s protocol. Isolated DNA was used to obtain TERTp and CTNNB1 exon 3 mutation profiles. Mutations were analyzed in tumor and non-tumor adjacent tissue. PCR was carried out in 25-μl volume containing 40 ng DNA,0.1 mM dNTP, 2.5 mM Mg2+ and 0.11 μM of each primer and 1 unit Taq polymerase (Taq DNA polymerase, Top-Bio) and 5% glycerol for TERTp. For CTNNB1, 20 ng of DNA, 0.2 mM dNTP, 2.5 mM Mg2+ and 0.2 μM of each primer and 1 unit Taq polymerase was used. TERTp and CTNNB1 amplification PCR was performed under following conditions: 95 °C for 5 min/3 min, next 33 cycles of 95 °C for 30 sec, 68 °C/60 °C for 1 min, 72 °C for 45 sec respectively. Primer pairs were described previously [23, 24]. Sanger sequencing was performed in both directions. Chromatograms were analyzed by visual comparison and mutation was called when the height of the over-lapping peak encompassed at least 33% of the primary peak.

### Polymorphism rs2853669 genotyping

SNP rs2853669 at TERTp was genotyped with TaqMan® SNP Genotyping Assays (Applied Bioscience) and 20 ng of isolated DNA on CFX96 Touch Deep Well Real-Time PCR Detection System (Bio-Rad). Genotyping conditions were as followed: 95 °C for 10 min, next 50 cycles of 95 °C for 15 sec and 60 °C for 1 min.

### Histology and immunohistochemistry

For each patient FFPE tissue blocks containing center of the tumor (TC) and invasive margin (IM) and, when available, separate block with non-tumor liver, 2- to 3-cm distant from the tumor site, were retrieved from the pathology archive; the detailed procedure are described elsewhere [25]. One or two tis-sue sections of 4-μm thickness were cut and mounted onto BOND Plus microscope slides (K8020, Leica Bio-systems Newcastle Ltd., United Kingdom). Immunoperoxidase detection of CD8+ T cells was performed using fully automated BOND-III IHC/ISH stainer with anti-CD8 monoclonal primary antibodies (clone 4B11) from Leica Biosystems (Newcastle Ltd., United Kingdom) and horseradish peroxidase (HRP)-linker antibody conjugate system (Bond™ Polymer Refine Detection). Sections were counterstained with Mayer’s hema-toxylin and embedded into Micromount mounting medium (Leica). Appropriate positive (tonsils) and negative tissue control samples were used. All sections were examined under Olympus CX41 microscope (Olympus, Tokyo, Japan) by two pathologists (AT and LC). Sections were microanatomically divided into TC and IM and non-tumor liver. The IM was defined as a 1000 μm-region centered on the border separating the malignant cell nests from the host tissue (Hendry et al. 2017), whereas TC represents the remaining tumor area [26]. Eight fields of view were selected by systematic uniform random sampling from the TC and IM using the objective 20×. Pictures were captured by the PromiCam 3-3CP digital camera (Promicra, Prague, Czech Republic), coupled with the QuickPhoto Industrial 3.2 software (Promicra, Prague, Czech Republic). The stereological analysis was performed by AT, who was blinded to the clinical outcome, using the computer assisted stereology software Ellipse (ViDiTo, Kosice, Slovak Republic). CD8+ nucleated cell profiles were counted using a probe consisting of a set of 2D unbiased counting frames (UCF). The number of CD8+ profiles per mm^2^ of area (Q_A_ = numerical density) was calculated as the number of positive lymphocytic profiles divided by the total, investigated counting frame area. To eliminate skewness in the distribution, the raw density of CD8+ nucleated cell profiles CD8+ (further denoted as density) was converted into corresponding percentile values and categorized into Low (below 25th percentile) vs Intermediate-High (25th–100th percentile) (further denoted as high).

### Statistical analysis

Statistical analysis was performed in the R environment (v.4.1.1). Spearman correlation was used to evaluate relations between variables. Survival analysis was performed with the Finalfit package [27]. Influence of individual and combined variables on TTR, DFS, OS was assessed by univariate and multivariable Cox regression. TTR was defined as period from tumor resection to recurrence/metastasis diagnosis. DFS was described as time from tumor resection until recurrence/metastasis detection or death due to any cause. OS was determined as the time from tumor resection to death due to any cause. Kaplan-Meier analysis was performed with the survival package and plots were generated with the survminer package [28, 29]. Differences between groups were assessed with Log-rank test. CD8+ densities in TC and IM were compared with the Wilcoxon test. Results were considered statistically significant when *p*-value < 0.05.

## Results

### TERT promoter and CTNNB1 mutations and polymorphism rs2853669

We sequenced 67 HCC cases for TERTp and CTNNB1 mutation analysis. Good quality sequences were obtained for 58 and 59 cases, respectively. We detected TERTp mutation in 44 (75.8%) patients. TERTp mutations were located in two mutually exclusive hotspots − 124 bp in 41 and − 146 bp in three cases, all of them were heterozygous changes. CTNNB1 mutations were identified in 21 patients (35.6%): 20 heterozygous and 1 homozygous. Of them 16 patients had one missense mutation, one had a missense mutation accompanied by a synonymous change, one had three missense mutations and two has only a synonymous change (Supplementary Table 1). Mutational changes in TERTp and CTNNB1 were not observed in adjacent non-tumor tissue. Additionally, all patients were successfully genotyped for polymorphism rs2853669. Overall frequencies for AA, AG and GG genotypes were 44.8, 32.8 and 22.4%, respectively. For most of our genetic and clinical data, we did not find any correlations. However, correlations were found between − 146 bp TERTp mutation and concentration of alfa-fetoprotein (*p* = 0.004), and between CTNNB1 mutations and diabetes mellitus (*p* = 0.004) and extent of encapsulation (*p* = 0.03) (Supplementary Table 2). We did not detect any correlations between mutations and the polymorphism. Clinical data were discussed in more detail in our previous work (25); only TNM stage (HR = 1.5. *p* = 0.032) and age (HR = 0.96, *p* = 0.007) had impact on patients TTR.

### CD8+ distribution

**Fig. 1:**
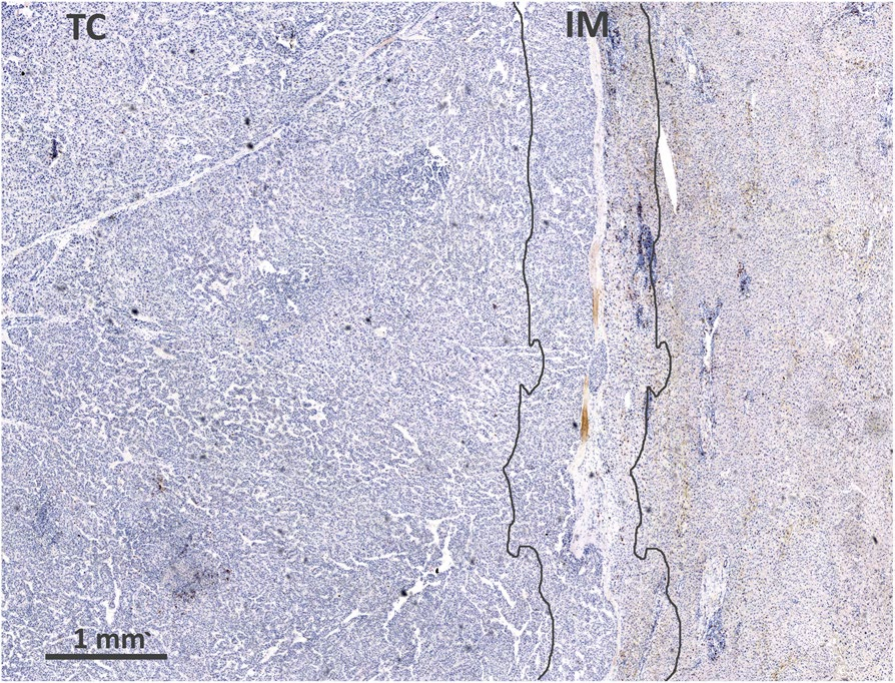
Immunoperoxidase staining for CD8+ lymphocytes in HCC. Regions of interest are denoted: TC (tumor center) and IM (invasive margin). The IM was defined as a 1-mm region centered on the border separating the malignant cell nests from the host tissue. The TC represented the remaining tumor area. Scale bar 1000 μm

IM displayed significantly higher density of CD8+ cells than TC (*p* < 0.0001). CD8+ cell densities in non-tumor liver were higher compared with TC (*p* < 0.0001), and lower when compared with IM. (Fig. 1., Table 2). Significant correlations between clinical data and CD8+ cells are presented in Supplement (Supplementary Table 3).

**Table 2:** Density of CD8+ cell (number of nucleated cell profiles/mm^2^)

Tumor center (*n* = 67)	Invasive margin (*n* = 66)	Non-tumor liver (*n* = 43)
44 (2–1475)	174 (31–1256)	109 (23–400)

### Prognostic values of CTNNB1, TERTp mutation, rs2853669 and CD8+ cell densities

**Fig. 2:**
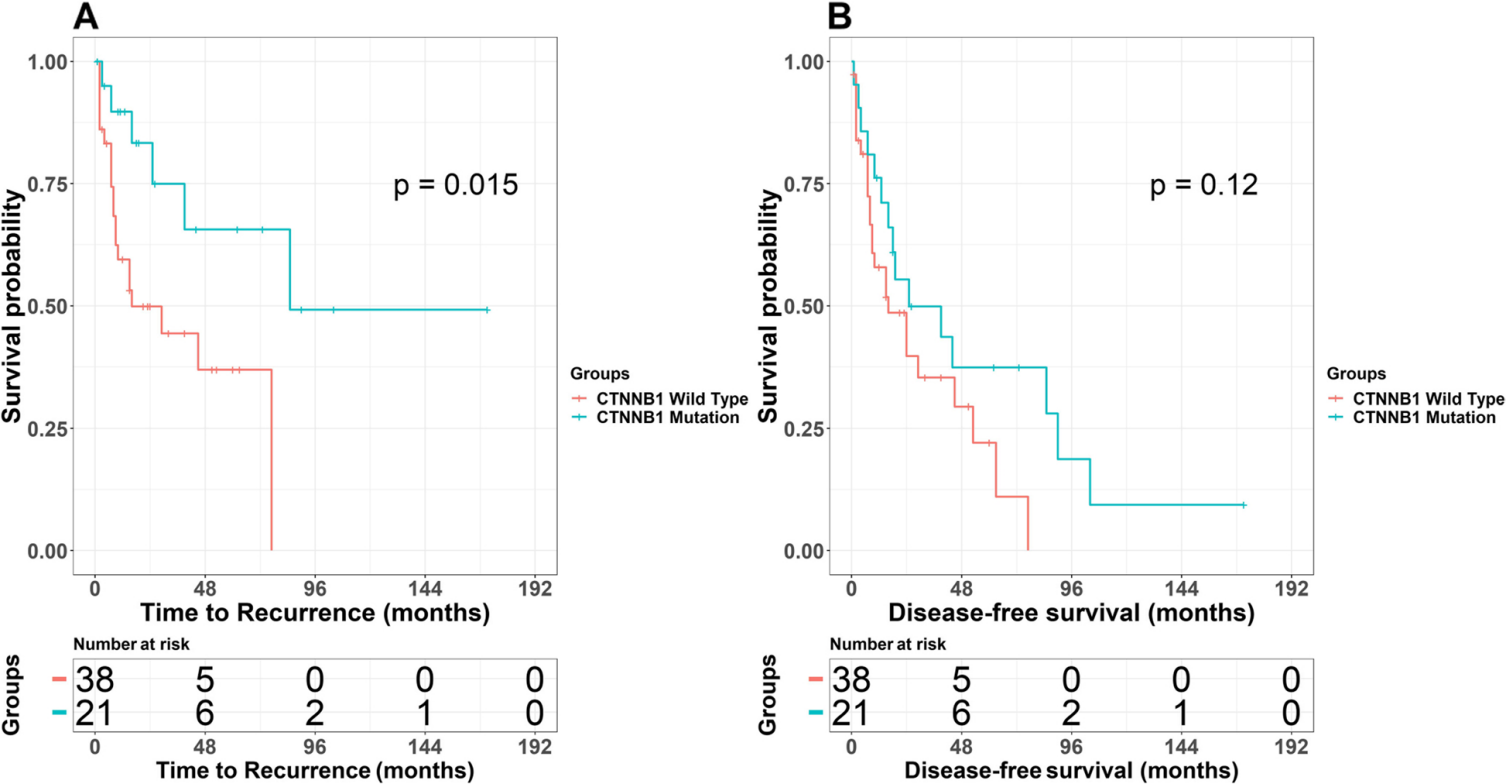
Kaplan-Meier analysis for TTR (**A**) and DFS (**B**) according to presence or absence of CTNNB1 mutation

We performed univariate Cox regression and Kaplan-Meier analysis to investigate the association between genetic, immune factors and TTR, DFS, OS. To confirm differences between groups we performed Log-rank test (Supplementary Table 4). None of the variables were associated with OS. We did not detect any associations between TERTp mutations and TTR or DFS. Presence of a CTNNB1 mutation was associated with longer TTR but did not affect DFS (Table 3, Fig. 2). Presence of homozygote GG in rs2853669 polymorphism was associated with longer TTR and longer DFS, whereas heterozygotes were not associated (Table 3, Supplementary Fig. 1). High CD8+ cell densities in TC and IM were associated with lower risk of recurrence and longer DFS. (Table 3, Supplementary Fig. 2).

**Table 3:** Univariable analysis of genetic and immune variables associated with TTR and DFS

Variable			TTR	DFS
	Group	N	HR	HR
TERTp mutation (124 bp)	WT	17	1.00	1.00
	MT	41	0.62 (0.27–1.40, *p* = 0.248)	0.72 (0.37–1.40, *p* = 0.335)
TERTp mutation (146 bp)	WT	61	1.00	1.00
	MT	3	1.88 (0.44–8.02, *p* = 0.392)	2.00 (0.61–6.55, *p* = 0.252)
CTNNB1 Mutation	WT	38	1.00	1.00
	MT	21	**0.31 (0.11–0.83,** ***p*** **= 0.020)**	0.57 (0.28–1.16, *p* = 0.121)
rs2853669	A/A	30	1.00	1.00
	A/G	22	0.73 (0.33–1.63, *p* = 0.443)	0.71 (0.36–1.40, *p* = 0.330)
	G/G	15	**0.29 (0.09–0.88,** ***p*** **= 0.028)**	**0.42 (0.20–0.90,** ***p*** **= 0.025)**
CD8 TC	LD	16	1.00	1.00
	HD	49	**0.32 (0.14–0.71,** ***p*** **= 0.005)**	**0.41 (0.21–0.80,** ***p*** **= 0.009)**
CD8 IM	LD	16	1.00	1.00
	HD	48	**0.34 (0.15–0.78,** ***p*** **= 0.011)**	**0.37 (0.19–0.73,** ***p*** **= 0.004)**

### Combined genetic and immune variables and their prognostic value

**Fig. 3:**
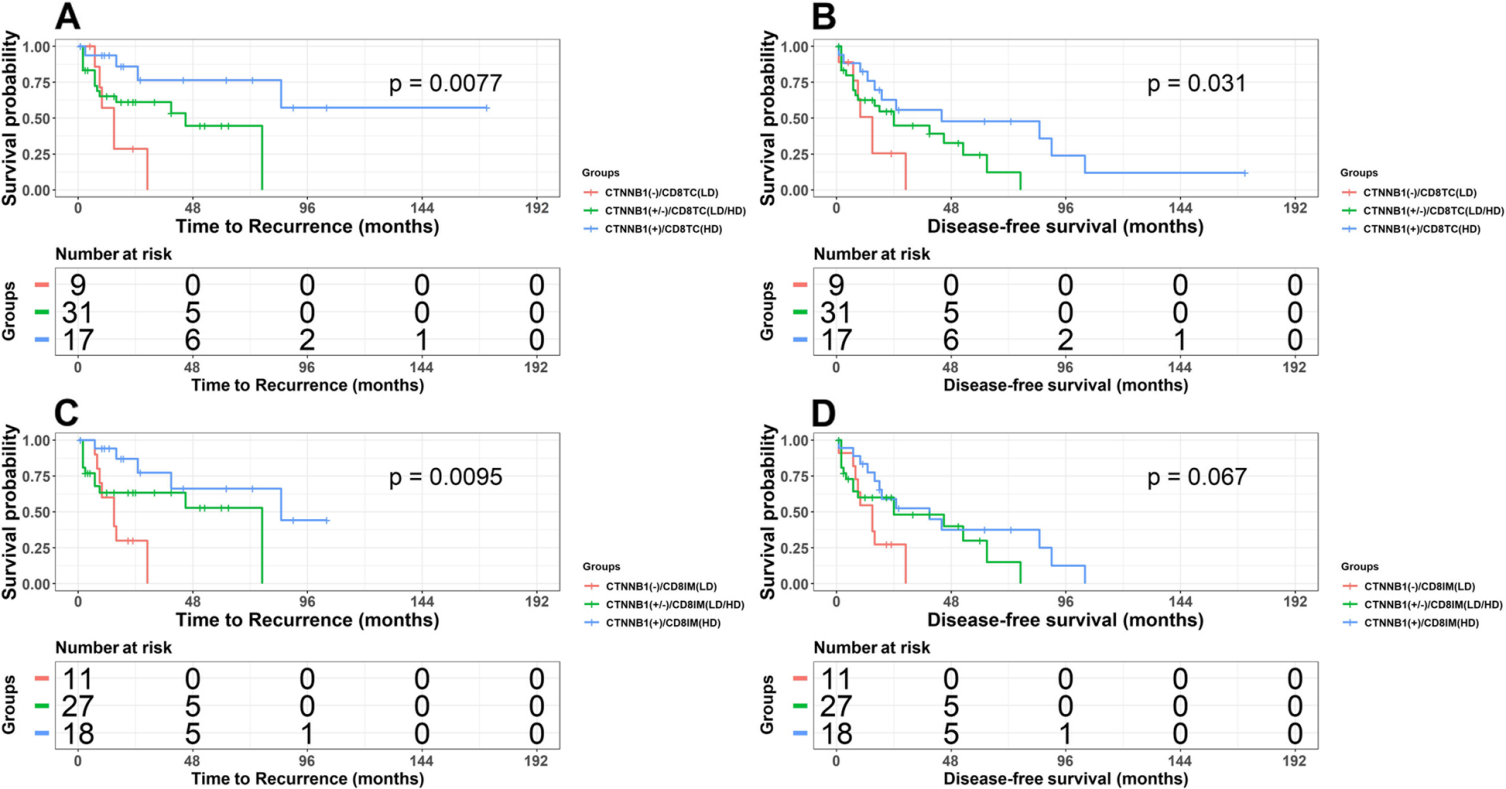
Kaplan-Meier analysis for TTR according to presence/absence of the CTNNB1mutation and high/low CD8+ densities in TC (**A**) and IM (**C**) and DFS in TC (**B**) and IM (**D**)

Next, we analyzed associations of combined genetic and immune factors with TTR, DFS and OS. The variables were grouped as follows: presence or absence CTNNB1 mutation, presence of AA, AG, or GG genotype for rs2853669 with low or high density of CD8+ cells. Altought TERTp mutations were not statistically significant in our individual analysis, due to previous reports [15, 30] we decided to combine them with polymorphism rs2853669 and evaluate their impact on patient’s outcomes. Individual analyses of CTNNB1 mutation and CD8+ cells density showed that the presence of mutation and high cell densities (HD) could be considered a favorable prognostic factor. Thus, we decided to analyze their combined influence on TTR and DFS. Cases were divided in 3 groups: presence of two good prognostic factors (CTNNB1(+)/CD8 + (HD)), presence of only one (CTNNB1(+/−)/CD8(LD/HD)) and presence of adverse prognostic factor (CTNNB1(−)/CD8(LD)). Presence of mutation in CTNNB1 and high CD8+ cells densities in TC and IM were associated with lower risk of recurrence (HR = 0.12 *p* = 0.005 and HR = 0.17 *p* = 0.012 respectively) and longer disease-free survival (HR = 0.25, *p* = 0.005 and HR = 0.34 *p* = 0.025 respectively) (Table 4 and Fig. 3).

**Table 4:** Analysis for TTR and DFS for combined genetic and immune variables

Variables			TTR	DFS
	Group	N	HR	HR
CTNNB1 Mutation + CD8+ (TC)	CTNNB1(−)/CD8TC(LD)	9	1.00	1.00
	CTNNB1(+)/CD8TC(HD)	17	**0.12 (0.03–0.52,** ***p*** **= 0.005)**	**0.25 (0.09–0.74,** ***p*** **= 0.012)**
CTNNB1 Mutation + CD8+ (IM)	CTNNB1(−)/CD8TIM(LD)	11	1.00	1.00
	CTNNB1(+)/CD8TIM(HD)	18	**0.17 (0.05–0.59,** ***p*** **= 0.005)**	**0.34 (0.13–0.87,** ***p*** **= 0.025)**
rs2853669 + CD8+ (TC)	AA/CD8TC(LD)	5	1.00	1.00
	AA/CD8TC(HD)	23	0.51 (0.14–1.92, *p* = 0.319)	0.53 (0.17–1.64, *p* = 0.271)
	GG/CD8TC(LD)	3	0.91 (0.15–5.46, *p* = 0.913)	1.00 (0.22–4.52, *p* = 0.995)
	GG/CD8TC(HD)	12	**0.10 (0.02–0.62,** ***p*** **= 0.013)**	**0.19 (0.05–0.69,** ***p*** **= 0.011)**
rs2853669 + CD8+ (IM)	AA/CD8TIM(LD)	8	1.00	1.00
	AA/CD8TIM(HD)	19	0.75 (0.24–2.33, *p* = 0.614)	0.69 (0.28–1.70, *p* = 0.416)
	GG/CD8TIM(LD)	2	1.07 (0.12–9.29, *p* = 0.953)	0.69 (0.09–5.59, *p* = 0.728)
	GG/CD8TIM(HD)	13	**0.17 (0.04–0.71,** ***p*** **= 0.015)**	**0.22 (0.08–0.61,** ***p*** **= 0.003)**
TERTp mutation (−124 bp) + rs2853669	TERTp(−)/AA	8	1.00	1.00
	TERTp(+)/AA	17	0.38 (0.12–1.16, *p* = 0.089)	0.43 (0.16–1.15, *p* = 0.092)
	TERTp(−)/GG	5	**0.08 (0.01–0.72,** ***p*** **= 0.024)**	**0.23 (0.06–0.82,** ***p*** **= 0.024)**
	TERTp(+)/GG	10	**0.15 (0.03–0.62,** ***p*** **= 0.009)**	**0.20 (0.06–0.64,** ***p*** **= 0.007)**

Similar to mutation in CTNNB1, the GG genotype in SNP rs2853669 can also be considered a “good” prognostic factor (Table 3). Therefore, we combined it with CD8+ cell densities in TC and IM. We observed statistically significant association between combination of density CD8+ with the GG genotype with TTR in TC and IM (HR = 0.10, *p* = 0.013 and HR = 0.17, *p* = 0.015, respectively) and with DFS in TC and IM (HR = 0.19, *p* = 0.011 and HR = 0.22, *p* = 0.003, respectively). Additionally, combination of CD8+ cell densities and the AG heterozygote in IM was associated with longer DFS survival (HR =0.36, *p* = 0.037). These findings were con-firmed in Kaplan-Meier analysis. (Fig. 3 and Supplementary Fig. 3) Combination of TERTp mutation (− 124 bp position) and polymorphism rs2853669 (where GG genotype can be considered as “good” prognostic factor) revealed that there was statistically significant association between GG genotype and lack of TERTp mutation and longer TTR (HR = 0.08, *p* = 0.024) and longer DFS survival (HR = 0.23, *p* = 0.024). Similar pattern was observed for presence of TERTp mutation and GG genotype, showing longer TTR and longer DFS (0.15, *p* = 0.009 and 0.20, *p* = 0.007, respectively).

### Multivariable analysis for individual and combined variables

Multivariable Cox regression models for each individual and combined parameters adjusted for TNM staging and age were built to assess their independent ability to predict TTR (Supplementary Table 5 and 6). Among the individual parameters, CTNNB1 mutation and rs2853669 were independently associated with TTR. Immune markers (CD8+ T cell densities in TC and IM) lost predictive capabilities after adjustment for TNM stage and age. Among combined parameters, combinations between rs2853669 and cell densities in TC and IM were associated with TTR. Moreover, combination of TERTp mutation and rs2853669 retain their predictive value after adjustment for TNM stage and age and was associated with longer TTR.

## Discussion

The novelty of the present study was that for the first time results were presented on the interplay be-tween genetic (CTNNB1 mutation and TERTp SNP rs2853669) and immune (CD8+ cells density) factors, their prognostic value and present usefulness of TTR in HCC prognostication. The GG genotype of SNPrs2853669, mutation in CTNNB1 and high CD8+ cell density were individually associated with improved TTR and DFS. Presence of CTNNB1 mutation and high CD8+ cell densities in both region of interest (TC or IM) strongly predicted longer TTR and DFS. Similarly, high CD8 + cell densities in TC or IM and presence of GG genotype were also associated with longer TTR and DFS. Combining positive genetic and immune factors additively improved each other’s influence.

TERTp mutations are a well-known cause of telomerase reactivation in many cancers [31]. We confirmed high frequency of TERTp mutation in the present HCC, not associated with viral hepatitis patients (75.8%). Our mutation frequency is somewhat higher than has been reported for another European dataset (56.6%) for no-viral HCC [32]. In concordance with available data, the nucleotide change in − 124 bp was the most common TERTp mutation (93.2%). TERTp mutation and polymorphism rs2853669 did not show any correlation with clinical and pathological data in our analysis. Neither did we observe any associations with TTR, DFS or OS for the TERTp mutation; the relation with prognosis is rather controversial, some literatureshowing association of TERTp mutation with poor DSF and OS [33] other does not [34, 35]. Our data showed a statistically significant association of GG genotype of SNP rs2853669 with longer TTR (HR = 0.29, *p* = 0.02) and longer DFS (HR = 0.42, *p* = 0.025). The associations were not significant for the AG heterozygotes. Presence of G allele modulated effect of TERTp mutations because the SNP destroys the pre-existing binding site for transcription factors EST [36]. Our data on the association of this SNP with favourable patient outcome are in agreement with prognostic data for various cancers [37]. Nevertheless, even deviant results have been reported for unknown reasons in an Asian population [15]. Previous reports which evaluate impact of TERTp mutation and rs2853669 and their combined effect on patient survival are not in agreement showing no correlation with survival or correlation with poor prognosis [15, 30]. Our data show opposite pattern where combination of GG allele and lack/presence of TERTp mutation is significantly correlated with longer TTR and longer DFS. We speculate firstly, that our patient etiology differed from the previous reports on viral etiology. Secondly, our data show a lesser role for a TERTp mutational status compared to higher predictive values for the rs2885669 GG allele.

The CTNNB1 gene is a key partner of the Wnt/β-catenin signaling pathway, which is activated in 30–50% of HCC cases [2]. This gene is one of the most frequently mutated cancer driver genes in HCC [37]. CTNNB1mutations are missense mutations, most of which are located at the exon 3 phosphorylation sites and they increase the expression of β-catenin protein in the cytoplasm and nucleus [17, 38]. According to the literature, nuclear expression is associated with improved prognosis, opposite to cytoplasmic and membranous expression, which are associates with poorer prognosis [39, 40]. CTNNB1mutations have been identified preferentially in early stages and HCV-related HCC [41, 42]. Our observed frequency of CTNNB1 mutations (35.6%) and the affected nucleotide sequences (Supplement Table 1) agreed with the literature (17). Available data show associations of CTNNB1 mutation with favorable prognosis (especially with longer OS) [19]. Our data showed no association with OS or DFS, but we observe longer TTR (HR =0.31, *p* = 0.02). Moreover, we found no association between CTNNB1 mutations and clinical or pathological data, which may be related to the non-viral etiology of the present patients compared to the majority of data originating from Asian populations with viral etiology.

TILs (T cells, B cells, natural killer (NK) cells) represent one of the elements of host immune response to tumor [5]. They may manifest pro- or anti-tumor characteristics dependent on lymphocyte subsets and their phenotypic orientation [43]. The role of TILs has been extensively explored in HCC [4, 5, 43, 44]. These results show that patients with high numbers of NK cells, B-cells, T-lymphocytes and dendritic cells had a favorable prognosis while poor prognosis was associated with high numbers of monocytes, neutrophils and Tregs [44]. Role of high CD8+ cell densities has been explored in many cancers, including HCC, usually showing associations with improved prognosis [4, 45–47]. In HCC, CD8+ cells were among the prognostic factors considered in the meta-analysis by Xu et al., showing associations of CD8+ densities in tumor and its margin with higher OS and DFS [4]. As high density of CD8+ cells predicts a favorable OS and DFS they may be used as biomarker of patient prognosis. On par with available data, our CD8+ analysis also showed high predictive value for DFS (HR = 0.41, *p* = 0.009), but not OS [48]. In our research, we also observed influence of high density of CD8+ cells on longer TTR (HR = 0.32, *p* = 0.005).

Many analyses have been published showing association with DFS or OS, but we did not find any data considering TTR. We believe that among the survival parameters analysed by us, TTR is highly relevant for prognostication. TTR is a tumor-specific outcome in contrast to less specific endpoints DFS and OS.

Previous prognostic studies have either used genetic or immune cell markers, but we are not aware of previous studies combining the two. There is a scientific rationale for the combination, because mutations are present in the tumor and immune response is mounted by the tumor microenvironment. These may be viewed as independent predictors of outcome although they interact, and mutation frequency may generally stimulate immune response [49, 50]. We decided to explore combined effect of CTNNB1 mutation and CD8+ cells in TC and IM as the first scenario. The second scenario was to combine three observed genotypes in rs2853669 with CD8+ cell densities. In both scenarios, certain combinations of CD8+ cell densities with genetic alteration refined prognostic value of our results. Cases presenting β-catenin mutation and high CD8+ cell densities in TC/IM presented much longer TTR (HR = 0.12, *p* = 0.005/HR = 0.17, *p* = 0.005 respectively) and longer DFS (HR = 0.25, *p* = 0.01/HR = 0.34, *p* = 0.02) than cases presenting only one of these “good” prognostic factors (Table 3 and Fig. 2). Similar to the first scenario, the GG genotype in SNP rs2853669 combined with high density of CD8+ cells presented longer TTR and longer DFS in both tumor regions of interest (Table 3). We could demonstrate that the combination of genetic and immune variable presents a much better prognostic value than individual variables. Multivariable Cox regression was performed for both individual and combined factors. Some individual and combined factors lost their significance after adjustment. We believe that was provoked by size of our group thus, our multivariate results are not reliable.

Our study presents few limitations, the foremost being the low case number. The case numbers limit particularly our analysis for combined factors. Thus, our present data cover only a small segment of genetic and immune landscape of tumor microenvironment. Nevertheless, we believe that a follow up study performed on greater number of cases and exploring different genetic alterations and different types of immune cells may further refine the prognostic tools. These data could be invaluable in treatment decision making.

## Conclusion

Our results show that factors both in the tumor and its environment influence HCC survival. The best predictive values for TTR were obtained by combining data for CTNNB1 mutations and CD8+ cell densities. Although we were able to analyze the most common somatic mutations in HCC, we were able to use only one immune cell marker, CD8+ cells, in this first analysis validating the integrative approach. In the future, many more cell types of the tumor microenvironment need to be tested in combination with the genetic markers. Moreover, it seems that combination of TERTp mutation (position − 124 bp) and rs2853669 may be a separate predictive factor for patients with non – viral etiology of HCC. The results, although based on small numbers of resected patients, are encouraging for study further integrative approaches for HCC risk stratification.

## Supplementary Information

**Additional file 1.**

## Data Availability

The datasets generated and analyzed during the current study are available in the Open Science Forum repository. Data can be accessed openly from the link: https://osf.io/2rwkj/.
